# Epidemiology of Infectious Pathogens in Horses with Acute Respiratory Disease, Abortion, and Neurological Signs: Insights Gained from the Veterinary Surveillance System for Horses in The Netherlands (SEIN)

**DOI:** 10.3390/vetsci12060567

**Published:** 2025-06-10

**Authors:** Kees van Maanen, Linda van den Wollenberg, Tara de Haan, Thibault Frippiat

**Affiliations:** 1 Royal GD, 7418EZ Deventer, The Netherlands; l.v.d.wollenberg@gddiergezondheid.nl (L.v.d.W.); t.d.haan@gddiergezondheid.nl (T.d.H.); 2 Sporthorse Medical Diagnostic Centre, 5384RC Heesch, The Netherlands; frippiat@sporthorsemdc.com

**Keywords:** equine influenza, equine herpesvirus, strangles, *Streptococcus equi*, epidemiology, microbiology, veterinary medicine, early warning, surveillance, horses

## Abstract

Monitoring infectious diseases is essential for safeguarding equine health and ensuring the sustainability of the horse industry. In 2019, the Royal Veterinary Association of the Netherlands (KNMvD) and Royal GD (GD Animal Health) launched SEIN (Surveillance of Equine Infectious diseases in the Netherlands), a voluntary surveillance system for laboratory-confirmed outbreaks of equid alphaherpesvirus 1 (EHV-1), equid alphaherpesvirus 4 (EHV-4), equine influenza virus (EIV), and *Streptococcus equi* subsp. *equi*. This retrospective study analyzed 364 confirmed outbreaks reported through SEIN between June 2019 and April 2023. *S. equi* was the most commonly reported pathogen overall (64%). Among outbreaks involving respiratory disease, *S. equi* accounted for 74% of cases, followed by EHV-4 (16%), EIV (6%), and EHV-1 (4%). The geographical distribution of outbreaks covered 80 of the 90 postal code regions (89%), and approximately half of all participating practices generated at least 1 alert. Vaccination data revealed low coverage against EHV-1/4, EIV, and *S. equi* among both affected horses and premises. Clinical signs overlapped between pathogens, but some were more pathogen-specific, e.g., coughing in EIV, and abscessation in *S. equi*. The SEIN system provided spatiotemporal information on confirmed outbreaks. These results underscore the importance of quick diagnostics and structured surveillance systems in guiding prevention strategies.

## Simple Summary

Infectious diseases, such as influenza, rhinopneumonitis, and strangles, can have a major impact on the health of horses and disrupt equestrian activities. To help monitor and control such diseases, a surveillance program called SEIN (Surveillance of Equine Infectious diseases in the Netherlands) was launched in 2019. In this study, we analyzed four years of outbreak data voluntarily reported by Dutch veterinarians. The results show that strangles, caused by the bacterium *Streptococcus equi* subsp. *equi*, accounted for the majority of cases, followed by rhinopneumonitis, caused by equid alphaherpesviruses 1 and 4, and influenza. Most affected horses were not vaccinated. The system helped identify patterns in disease spread over time and across regions. It also provided information on clinical signs and the vaccination status of affected horses. These findings highlight the importance of disease surveillance, quick diagnostics, and vaccination to protect horse health and reduce the impact of outbreaks on the equine sector.

## 1. Introduction

Infectious diseases pose significant health risks to equine populations, with outbreaks potentially leading to severe health consequences, economic losses, and disruption to equine activities [1,2]. Monitoring these outbreaks is critical for preventing their spread and minimizing their impact on horse health. An outbreak is defined as the occurrence of cases of a particular disease in a population or geographic area. Several infectious diseases are of particular concern in equine populations due to their contagious nature, clinical severity, and potential for outbreaks. Common respiratory infectious diseases in horses include equine rhinopneumonitis, equine influenza, and strangles.

Equine rhinopneumonitis is caused by either equid alphaherpesvirus 1 (EHV-1, formerly equine herpesvirus 1) or equid alphaherpesvirus 4 (EHV-4, formerly equine herpesvirus 4). These alphaherpesviruses are enveloped DNA viruses belonging to the *Orthoherpesviridae* family. EHV-1 can cause respiratory disease, abortion, neonatal death, and neurological disease, the latter referred to as equine herpesvirus myeloencephalopathy (EHM). EHV-4 is generally associated with respiratory symptoms but can also incidentally cause abortion [3]. Equid alphaherpesviruses are spread by direct contact, respiratory secretions, and fomites. In addition, EHV-1 can also be transmitted through exposure to contaminated aborted fetuses, placentas, and vaginal fluids [4]. EHV-1 outbreaks, particularly of the neurological form (EHM), can be a serious concern, with high morbidity and potential mortality [5].

Equine influenza is caused by the H7N7 (considered extinct) and H3N8 subtypes of the equine influenza virus (EIV), an RNA virus of the *Orthomyxoviridae* family. EIV is highly contagious and is transmitted via aerosols from infected horses, especially during coughing, and through contact with contaminated surfaces. Clinical signs include fever, nasal discharge, coughing, lethargy, and loss of appetite. Outbreaks can spread rapidly within equine populations, particularly in unvaccinated horses, resulting in significant economic losses and disruptions to competitions [6].

Strangles is caused by *Streptococcus equi* subsp. *equi*, a Gram-positive, beta-hemolytic bacterium of the *Streptococcaceae* family. *S. equi* is highly contagious and primarily transmitted through direct contact between horses or indirect contact with contaminated surfaces such as water troughs, feed buckets, and grooming equipment. The bacteria are shed in nasal secretions and in pus from ruptured abscesses. *S. equi* can survive in the environment for several weeks, especially in water and under moist conditions [7], facilitating the spread of the disease through contaminated fomites, especially in crowded settings such as stables or during transport. Clinical signs include lymphadenopathy, abscess formation, fever, and nasal discharge [8].

Epidemiologic surveillance is critical in veterinary health strategies aimed at monitoring, preventing, and controlling outbreaks of infectious diseases in equine populations. It involves the systematic collection, analysis, interpretation, and dissemination of health data related to infectious diseases affecting horses. Effective surveillance is essential for early detection of outbreaks, informing response strategies, and guiding policy decisions to protect equine health and the equine industry as a whole. Several equine infectious disease surveillance programs exist in, among others, Belgium, France, Switzerland, the United Kingdom, and the United States of America [9,10,11,12].

In 2019, the Royal Veterinary Association of the Netherlands (KNMvD) and Royal GD (GD Animal Health) collaborated to develop a surveillance program for equine infectious diseases in the Netherlands, known as Surveillance of Equine Infectious diseases in the Netherlands (SEIN). The purpose of this program was to create an early warning system that provides relevant spatiotemporal information to participating veterinary practices, enhances awareness of these infectious diseases, and collects data on the clinical signs and vaccination status of affected horses in confirmed outbreaks.

The present study aimed to retrospectively analyze the outbreaks of acute respiratory disease, abortion, and neurological signs caused by the equine infectious pathogens EHV-1, EHV-4, EIV, and *S. equi*, as reported through SEIN from June 2019 to April 2023, with a focus on spatiotemporal information relevant to the participants. We attempted to collect additional information to allow for descriptive epidemiological analysis of the reported outbreaks.

## 2. Materials and Methods

Between June 2019 and April 2023, licensed veterinary practitioners in the Netherlands were invited to participate in SEIN through communications from the KNMvD and Royal GD. Participating veterinary practitioners signed on behalf of their clients for consent to anonymously report outbreaks that occurred in the Netherlands and were confirmed by the GD laboratory. Participants were subsequently informed about these outbreaks at a two-digit postal code level (at this level, the Netherlands is divided into 90 areas).

For suspected respiratory disease, nasopharyngeal swabs were submitted for testing. For suspected strangles cases, various samples could be submitted, including nasopharyngeal swabs, nasopharyngeal lavages, guttural pouch lavages, and pus from abscesses. In most suspected EHM cases, both nasopharyngeal swabs and EDTA blood samples were submitted. In most cases of abortion or neonatal death, both a lung aspiration biopsy from the fetus/foal and a vaginal swab from the mare were submitted.

Samples were tested by real-time polymerase chain reaction (PCR). RNA and DNA were extracted from the different samples used in this study with the MagMax RNA/DNA isolation kit on a KingFisher^TM^ Flex Purification System (Thermo Fisher Scientific, Waltham, MA, USA). Real-time PCR, as previously described, with some modifications, was used for the detection of EIV, EHV-1, EHV-4, and *S. equi* [13,14,15]. In brief, the extracted RNA/DNA was tested using the AgPath-ID^TM^ One-Step RT-PCR Kit (Thermo Fisher Scientific, Waltham, MA, USA) on a QuantStudio^TM^ 5 system (Thermo Fisher Scientific, Waltham, MA, USA) with the following program: 10 min at 45 °C, 10 min at 95 °C, followed by 45 cycles of 15 s at 95 °C, and 45 s at 60 °C.

When a sample tested positive for any of the targeted pathogens, and the veterinary practice was a SEIN participant, it was contacted for additional information about the outbreak. This included details on clinical signs, the number of affected horses on the premises, the total number of horses present, and the control measures implemented. Data were also collected regarding the vaccination status of the affected horses sampled for the detected pathogen(s), whether vaccination was up to date according to the manufacturer’s instructions, and whether other horses present on the premises were vaccinated. Reports were shared by email as soon as the information was complete, with a turnaround time of approximately 1–7 days. Confirmed EHV-1 outbreaks involving abortion or neurological signs were reported separately.

In June 2024, data were retrospectively retrieved from the Royal GD database and compiled in a Microsoft Excel sheet. Information on vaccine sales for the targeted pathogens was generously provided by Fidin (The Hague, Netherlands), an association representing Dutch manufacturers and importers of veterinary medicinal products. Fisher’s exact test was used for statistical analysis of the clinical data, using Prism 10.4.0 for macOS (GraphPad Software, Boston, MA, USA). To assess differences in monthly outbreak frequency, a Kruskal–Wallis test was performed, with post-hoc analysis using Dunn’s test with Bonferroni correction. Statistical significance was set at *p* < 0.05.

## 3. Results

### 3.1. Yearly Participation of Veterinary Practices

At the start of the SEIN program in June 2019, 43 veterinary practices were participating. By the end of 2019, this number had increased to 129. The number of participating practices continued to grow, reaching 165 by the end of 2020, 183 in 2021, 196 in 2022, and 201 by the end of April 2023. At the time of manuscript submission (May 2025), 246 veterinary practices were participating in SEIN.

### 3.2. Temporal Distribution of Outbreaks

**Figure 1:**
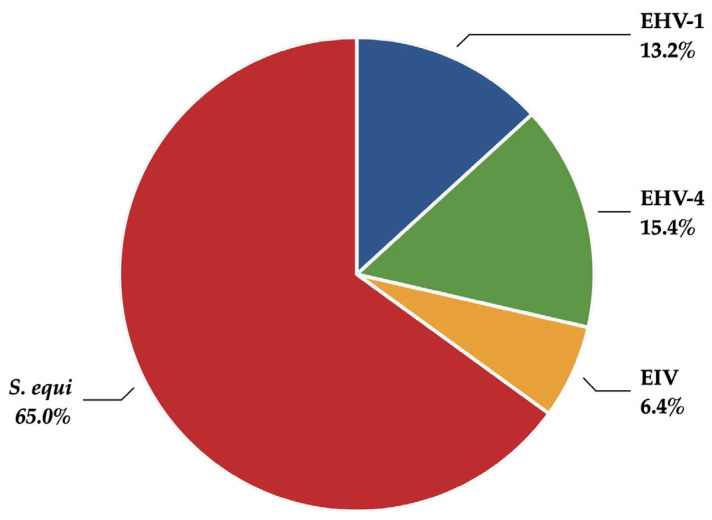
Distribution of pathogens reported through SEIN between June 2019 and April 2023. EHV-1: equid alphaherpesvirus 1; EHV-4: equid alphaherpesvirus 4; EIV: equine influenza virus; *S. equi*: *Streptococcus equi* subsp. *equi*.

Table 1 presents the annual number of reports per pathogen for confirmed outbreaks of the four pathogens monitored in SEIN. Additionally, outbreaks involving co-infections were identified and reported accordingly. A total of 364 reports were generated between June 2019 and April 2023, mainly caused by *S. equi* (Figure 1). When compared to the total number of outbreaks confirmed by the GD laboratory during the same period, 18% of EHV-1 outbreaks, 28% of EHV-4 outbreaks, 24% of EIV outbreaks, and 41% of *S. equi* outbreaks were reported through SEIN. This partial coverage was attributed to several factors, including the restriction that only analyses submitted by SEIN participants were included and that SEIN reports were generated only after additional information had been obtained.

Table 2 presents the annual apparent prevalence, as confirmed by real-time PCR, from all nasopharyngeal swabs submitted to the GD laboratory during the reporting period of SEIN described in this paper. It was not possible to differentiate between samples from horses with acute respiratory disease and those from clinically healthy horses, because providing this information was not obligatory, and therefore it was often missing from the submission forms. However, the latter category was considered small in this dataset, since the GD laboratory does not routinely test samples for export purposes. An exception occurred following the international EHM outbreak connected to the Sunshine Tour in Valencia in 2021, when healthy horses were screened by GD for EHV-1 as a prerequisite for participation in certain competitions, as mandated by the Fédération Equestre Internationale.

**Figure 2:**
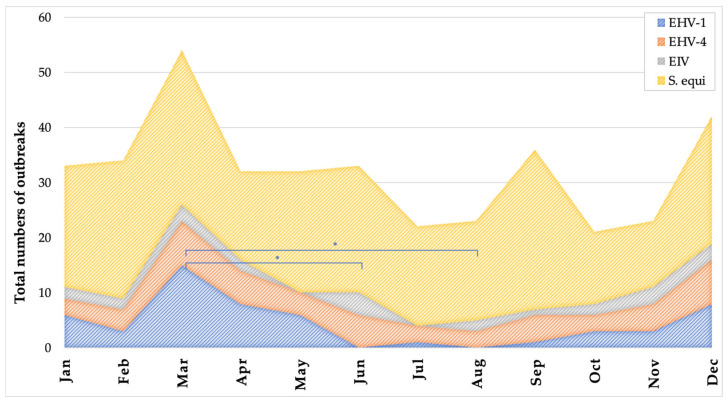
Monthly distribution of confirmed outbreaks caused by equid alphaherpesvirus 1 (EHV-1), equid alphaherpesvirus 4 (EHV-4), equine influenza virus (EIV), and *Streptococcus equi* subsp. *equi* reported through the SEIN surveillance system between June 2019 and April 2023. The total number of outbreaks is plotted per calendar month, aggregated over nearly four years, with one value for May missing. Asterisks indicate a statistically significant difference (*p* < 0.05), as determined by the Kruskal–Wallis test.

Figure 2 presents the monthly distribution of confirmed outbreaks per pathogen reported through SEIN between June 2019 and April 2023. Outbreak counts were aggregated by calendar month over the nearly four-year surveillance period (with one missing value for May). For each month, most outbreaks were attributed to *Streptococcus equi* subsp. *equi*, followed by EHV-1, EHV-4, and EIV. Visual inspection suggests a seasonal trend, with a peak in outbreak frequency during March for all pathogens. The number of EHV-1 outbreaks in March was significantly higher than in both June and August (*p* < 0.03). No other statistically significant differences were found between months for the other pathogens. These results indicate a possible seasonal pattern in EHV-1 activity, which may support the strategic timing of vaccination and increased vigilance during high-risk months.

### 3.3. Geographical Distribution of Outbreaks

**Figure 3:**
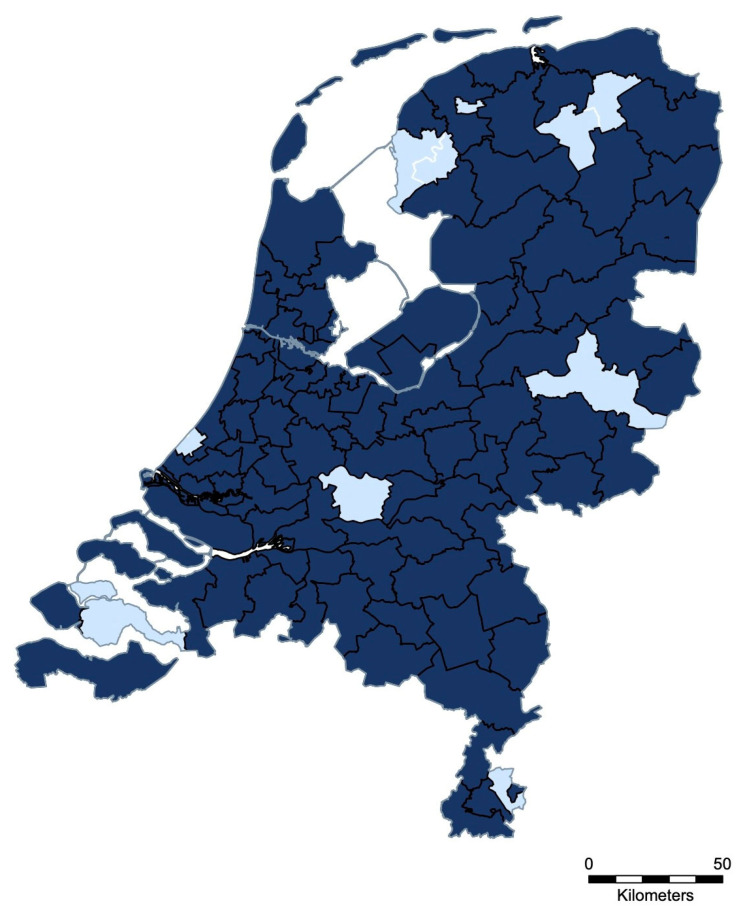
Geographical distribution of SEIN outbreaks in the Netherlands (June 2019–April 2023). Dark blue indicates two-digit postal code areas where at least one alert was generated. Light blue indicates areas with no reported outbreak.

Figure 3 presents the geographical distribution of SEIN reports from June 2019 to April 2023. Out of the 90 existing two-digit postal code areas in the Netherlands, 80 (88.9%) were represented by at least 1 report. At least 1 outbreak report was generated for 96 of the 201 participating veterinary practices (47.8%), with an average of 3 reports per practice and a maximum of 19 alerts submitted by a single practice during the whole period.

### 3.4. Clinical Syndromes Associated with EHV-1

In addition to respiratory clinical signs, EHV-1 can also induce the more serious sequelae of abortion/neonatal mortality and neurological signs known as EHM. Therefore, the EHV-1 confirmed outbreaks reported during the SEIN period were also categorized according to clinical presentation, as shown in Table 3.

### 3.5. Clinical Syndromes Associated with Outbreaks

#### 3.5.1. Acute Respiratory Disease

Table 4 presents all clinical signs reported in confirmed single-pathogen respiratory outbreaks of EHV-1, EHV-4, EIV, and *S. equi* outbreaks within SEIN (June 2019–April 2023). Most outbreaks involved *S. equi* (74.0%), followed by EHV-4 (15.9%), EIV (6.0%), and EHV-1 (4.1%). Submandibular and/or retropharyngeal lymphadenopathy was significantly more common in *S. equi* cases compared to EHV-1 (*p* = 0.002), EHV-4 (*p* = 0.003), and EIV (*p* = 0.048). Abscess formation was observed only in *S. equi* cases. In contrast, coughing was significantly less frequent in *S. equi* cases than in those caused by EHV-4 (*p* = 0.002) or EIV (*p* < 0.001).

#### 3.5.2. Abortion, Neonatal Mortality, and EHM

Clinical signs reported in the 22 outbreaks of abortion/neonatal mortality recorded within SEIN (June 2019–April 2023) included abortion (86.4%), neonatal mortality (13.6%), fever in the mare (4.5%), nasal discharge in the mare (4.5%), and stridor/dyspnea in the foal (4.5%). Among the 13 outbreaks of EHM, the reported clinical signs were ataxia (61.5%), fever (61.5%), tail hypotonia/paralysis (46.2%), limb edema (30.8%), bladder paralysis/distended bladder (23.1%), recumbency (15.4%), nasal discharge (7.7%), and depression/lethargy (7.7%).

One report of abortion/neonatal mortality involved EHV-4. In addition to neonatal mortality, the case included nasal discharge in the mare.

### 3.6. Vaccination Status for the Detected Pathogen(s)

#### 3.6.1. According to Participating Veterinary Practices

**Figure 4:**
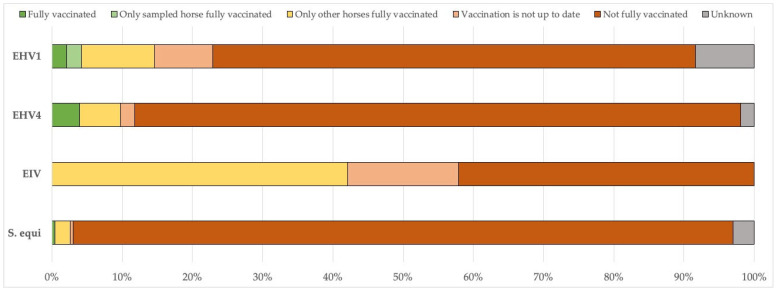
Vaccination status of the horses on premises with confirmed and reported outbreaks, excluding mixed infections. EHV-1: equid alphaherpesvirus 1; EHV-4: equid alphaherpesvirus 4; EIV: equine influenza virus; *S. equi*: *Streptococcus equi* subsp. *equi*.

Figure 4 shows the vaccination status of the horses on premises with confirmed and reported outbreaks, excluding mixed infections. The findings indicate that most of the sampled horses were not vaccinated against the detected pathogen(s), nor were the other horses on the premises. For equine influenza, none of the horses with a confirmed EIV diagnosis had an up-to-date vaccination history, while 42% of the other horses on the premises had been vaccinated.

#### 3.6.2. According to Vaccine Sales

**Figure 5:**
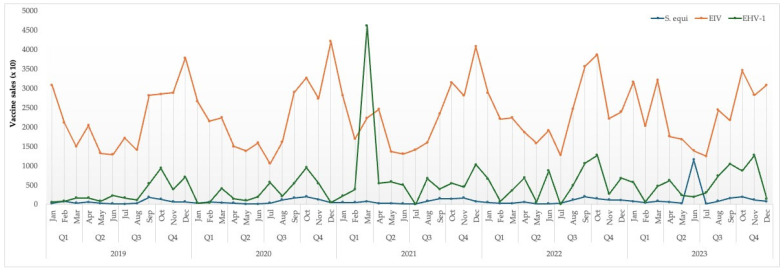
Quarterly sales data of vaccines for *Streptococcus equi* subsp. *equi* (*S. equi*), equine influenza virus (EIV), and equid alphaherpesvirus 1 (EHV-1) in the Netherlands between 2019 and 2023 (with the courtesy of Fidin).

Figure 5 shows the temporal trend in equine vaccine sales for the pathogens of interest during the period June 2019 to April 2023. Sales of EIV vaccines show a consistent cyclical pattern, with recurring peaks typically observed in the first and fourth quarters of each year. These trends likely reflect standard biannual vaccination protocols, as well as the influence of seasonal management practices such as the transitions between stable and pasture seasons and competition schedules, which are divided into an indoor and outdoor season. Vaccine sales for EHV-1 were generally stable with occasional surges, most notably in early 2021. This spike coincides with the international EHM outbreak connected to the Sunshine Tour in Valencia in 2021, suggesting that heightened awareness and risk perception significantly influenced vaccination behavior during that period. Vaccine sales for *S. equi* remained consistently lower than for the other pathogens, with a notable peak observed in June 2023. Although the exact cause of this increase is unknown to the authors, it may reflect a growing awareness or implementation of preventive strategies against *S. equi* within the equine sector. Overall, these data underscore how seasonal factors, disease outbreaks, and vaccination guidelines shape the demand for vaccines against respiratory pathogens in horses.

## 4. Discussion

Monitoring equine infectious diseases is essential to safeguard horses’ health and welfare and promote the equine industry’s sustainability.

The goals of a surveillance system may include one or more of the following: monitoring disease trends over time, detecting changes in disease occurrence, responding to increases in numbers of cases above expected levels, detecting and monitoring outbreaks with respect to source, time, population and location, contributing to the evaluation and monitoring of prevention and control programs, identifying populations at risk and applying targeted preventative measures, evaluating new diseases, sources and modes of transmission, and monitoring changes to pathogens such antigenic drift and shift in influenza viruses [16]. The shift in the role of horses, from primarily livestock and working animals to more of a companion animal, and the diversity of their use (e.g., breeding, food production, competitive equestrian sport [17], traditional and cultural events, companion animals, and equine-assisted therapy [18]) resulted in a complex equine industry comprising numerous stakeholders with disparate interests. This makes the surveillance and control of diseases in equines particularly challenging [10].

Nevertheless, several countries have developed systems for notifying stakeholders of clinical and laboratory-confirmed cases of equine infectious diseases. European examples include the French “Réseau d’Epidémio-Surveillance en Pathologie Equine” (RESPE), the Belgian “Equi Focus Point Belgium” (EFPB), the Swiss Equinella, and the Dutch SEIN. Established in 1999, the RESPE service plays a central role in curbing the spread of equine pathogens in France. The number of RESPE-associated veterinary practices increased 2.5-fold from 2006 to 2010 (112 to 288), as reported by Legrand et al. [11]. The number of SEIN-associated veterinary practices also increased almost 2-fold from 2019 till the moment of submission of this paper (129 to 246). Equinella is a voluntary veterinary-based surveillance system of non-notifiable equine infectious diseases and clinical signs in Switzerland. Oszelik et al. evaluated Equinella by reviewing the reports submitted since its relaunch in November 2013 until April 2019 [10]. The coverage of Equinella was assessed to be 50.8% of the Swiss equine population. Over the 5.5 years, of all 102 registered veterinarians, 67 (65.7%) submitted at least 1 report. On average, these veterinarians submitted 1.7 reports per year. However, the reports of clinical disease in Equinella are not necessarily confirmed by laboratory testing (55% of all reports were accompanied by laboratory testing), as is the case for RESPE, EFPB, and SEIN. For SEIN, over nearly 4 years, at least 1 confirmed outbreak alert was generated for 96 veterinary practices, representing 47.8% of the registered equine veterinarians, with a mean of 3 alerts per practice. Furthermore, the geographical coverage of SEIN alerts over this period was high, as illustrated in Figure 2. Complementing these national efforts, the International Collating Centre (ICC), coordinated by Equine Infectious Disease Surveillance (EIDS), has been compiling outbreak reports across countries for more than 30 years [12].

The distribution of the pathogens investigated in the present study shows a clear predominance of *S. equi* infections (Table 1 and Figure 1), which was also reported by Oczelik et al. [10]. Identifying respiratory viral infections in horses can be inferred from clinical signs, including fever, cough, nasal discharge, and lethargy. However, these signs are not specific to viral infections. For example, strangles can exhibit similar clinical manifestations [8]. Our results also confirm that clinical signs, such as fever, nasal discharge, and lethargy, are common to multiple pathogens, although coughing was reported more frequently in EIV infections (Table 3 and Table 4). Pusterla et al. reported the results of a voluntary surveillance program for EIV in the USA from 2010 to 2013 [19]. One of the findings was that fever, nasal discharge, and coughing were positively associated with EIV-positive horses. For many of the confirmed strangles cases in our study, abscessation was not reported. This may be due to the early stage of the disease when the samples were collected in cases of abscessation and rupture into the guttural pouch of the retropharyngeal lymph nodes, or when a nonspecific disease course occurred in partially immune horses. Additionally, practitioners may not submit samples when they consider clinical cases with external rupture of lymph nodes highly suggestive of strangles.

Since EIV is highly contagious, infections with this pathogen typically spread more quickly within a horse population than other respiratory viruses or streptococci. On the other hand, certain respiratory viruses are associated with disorders that affect systems beyond the airways. For example, EHV-1 can cause neurological signs, and EHV-1 and EHV-4 have been associated with abortion, with a potentially highly negative impact on the daily routine of riding schools, racetracks, breeding farms, and veterinary hospitals (Table 2 and Table 4). This underscores the importance of preventing these pathogens from spreading within the equine population. Effective control and prevention of respiratory viral infections require optimal surveillance and rapid, accurate diagnosis of the causative pathogens [1].

Although the four pathogens reported in SEIN are generally considered primary pathogens, they may sometimes also be detected in samples from clinically healthy horses [1]. Pusterla et al. reported the detection frequency of respiratory viruses (EIV, EHV-1, equid gammaherpesvirus 2 [EHV-2], EHV-4, equid gammaherpesvirus 5 [EHV-5], equine rhinitis A virus [ERAV], equine rhinitis B virus [ERBV]), and bacteria (*S. equi* and *Streptococcus equi* subsp. *zooepidemicus*) in nasal secretions from 162 healthy sport horses [20]. Nasal swabs were collected at a single time point and analyzed using quantitative PCR (qPCR). The detection frequency of respiratory pathogens in nasal secretions was 38.9% for EHV-2, 36.4% for EHV-5, 19.7% for *S. zooepidemicus*, but only 1.2% for ERBV, 0.6% for *S. equi*, and 0.0% for EIV, EHV-1, EHV-4, and ERAV, corroborating the clinical relevance of a positive test result for the latter pathogens. Smith et al. demonstrated that equid gammaherpesviruses are particularly frequently detected in horses recently imported into the USA after long-distance travel [21]. The EHV-1 qPCR-positive horses all displayed clinical signs of respiratory disease. In contrast, qPCR-positive horses for EHV-2, EHV-4, and EHV-5 did not predictably exhibit clinical signs of respiratory disease. None of the horses were found to be qPCR-positive for EIV, ERAV, ERBV, or *S. equi*, which aligns with the results of Pusterla et al. [20].

For EIV, there was an exceptionally high level of occurrence in 2019, with a peak in the UK, where 229 outbreaks were recorded, and distributed throughout the year. This represented the highest annual incidence reported in all the years considered. Reports from France and Germany contributed importantly to the total number, with 52 and 29 outbreaks, respectively. In 2019, outbreaks were concentrated in the winter and spring, with a lower occurrence during the summer months, indicating a seasonal pattern. Countries such as Belgium, the Netherlands, and Ireland showed a moderate number of outbreaks, while Italy, Finland, and others recorded only sporadic cases [12]. According to these authors, the number of EIV outbreaks in the Netherlands was more stable than elsewhere, varying between two and eight cases per year. This suggests a moderate endemic situation, likely effectively managed through vaccination and surveillance programs. However, it should be noted that an epidemic of EIV/H3N8/FC1 had already begun in the Netherlands in December 2018, following years of minimal EIV activity in Northwestern Europe, with numerous outbreaks occurring in the spring of 2019. Since SEIN was started in June 2019, the initial phase of this epidemic was not reported within the SEIN dataset. Additionally, our vaccination data on premises with confirmed EIV outbreaks do not indicate a high vaccination coverage, nor a high proportion of horses with an up-to-date vaccination status (Figure 4).

Vaccination against EIV remains the most effective prevention strategy for limiting viral spread and reducing disease severity, as therapeutic options for treating viral infections are limited [22]. However, the duration of protection conferred by vaccination is limited and requires regular booster vaccinations to maintain an adequate level of immunity, thereby increasing the costs and resources needed. In addition, the antigenic evolution of the virus necessitates frequent updates to vaccine formulations to address emerging variants [12]. However, despite high EIV-specific immune coverage and the use of an EIV vaccine fully updated according to the last OIE recommendation on EIV vaccine strain composition (i.e., EIV vaccines should contain representative EIV strains of both FC1 and FC2 sub-lineages), clinical equine influenza cases were reported again in France in early December 2018. Sequencing results revealed that H3N8 EIV strains at the origin of the 2018–2019 epidemic belong to the FC1 sub-lineage, which has not been isolated in France since 2009 but was commonly found in North and South America [23]. It is imperative to note, however, that all available field and veterinary reports indicated that clinical signs of disease observed in EIV-vaccinated horses were clearly reduced compared with unvaccinated animals, reaffirming the benefit of EIV vaccination [19]. During the 2019 epidemic, we also observed that horses with severe clinical signs had generally received their last vaccination more than six months prior (van Maanen, unpublished data).

Our data also show very extremely low vaccination coverage for EHV-1, EHV-4, and *S. equi* among affected horses and premises with confirmed outbreaks. This might imply that well-vaccinated premises were protected against clinical disease, but we have no data to substantiate this assumption. Overall vaccination sales in the Netherlands also indicate low vaccination coverage for these pathogens in general. Our data indicate annual EIV vaccine sales ranging from 317,770 to 378,770 doses. The Dutch equine population is estimated to be approximately 450,000 horses, with some individuals requiring more than one vaccination per year, such as a primary vaccination series or biannual boosters.

SEIN is based on laboratory-confirmed outbreak alerts. However, relevant information was often missing from the sample submission forms, requiring the SEIN team to make considerable efforts to obtain the necessary data through follow-up phone calls, emails, and reminders. Equinella is based on an online reporting tool for clinical disease, where registered veterinarians must take the initiative to use the tool and report their clinical cases. To promote reporting via Equinella, non-monetary and monetary incentives are offered to registered veterinarians. These include a monthly electronic newsletter on national and international equine health events, one free professional veterinary education course per year, direct contact with experts from the Equinella team, a password-secured internal space within the online platform, containing specific disease information sheets, reduced fees for laboratory diagnostic testing, and a mobile phone messaging service in case of an equine infectious disease outbreak in Switzerland [10]. This strategy was not applied to SEIN, which is entirely based on voluntary surveillance.

In the meantime, after nearly four years, SEIN has undergone slight adaptation. The number of pathogens reported has been extended since April 2023, including *Rhodococcus equi*, *Salmonella enterica* spp., equine coronavirus, *Babesia caballi*, *Theileria equi*, and *Anaplasma phagocytophilum*. Outbreaks are now reported once a week in clusters of respiratory, gastrointestinal, and tickborne pathogens, and we no longer approach veterinary practices for additional information. This has led to a significant increase in the number of outbreaks reported by SEIN. Only in specific cases, such as EHM outbreaks, will we contact the veterinary practice concerned and report any additional information. Nevertheless, maintaining high participation requires continuous feedback between voluntary participants and the management team responsible for the surveillance system. This can be achieved by taking a more transdisciplinary approach to surveillance system management, including conducting regular feedback dialogues accompanied by tailored incentives [10].

Preventive strategies, such as vaccination and biosecurity, monitoring of diseases in equine populations, and quick and accurate diagnostics during outbreaks, remain the most effective instruments to limit the propagation and transmission of primary pathogens in the horse. These preventive measures should be supported by continuous education of stakeholders and veterinarians on the early clinical signs and transmission dynamics of key equine pathogens. Enhancing the participation of veterinarians and owners in passive surveillance systems, such as SEIN, is therefore a crucial objective to mitigate the further risk of disease outbreaks.

## Figures and Tables

**Table 1:** Number of reports per pathogen per year, as reported by the SEIN email alert system. EHV-1: equid alphaherpesvirus 1; EHV-4: equid alphaherpesvirus 4; EIV: equine influenza virus; *S. equi*: *Streptococcus equi* subsp. *equi*.

Year	EHV-1	EHV-4	EIV	*S. equi*	EHV-1 and EHV-4	*S. equi* and EHV-1	*S. equi* and EHV-4	*S. equi* and EIV	Total
2019 ^1^	7	8	2	29			1	1	48
2020	16	14	6	63			1	1	101
2021	13	13	5	79	1				111
2022	9	10	5	52		1	4	3	84
2023 ^2^	3	6	1	10					20
Total	48	51	19	233	1	1	6	5	364

^1^ June to December; ^2^ January to April.

**Table 2:** Prevalence of infectious pathogens detection in the nasopharyngeal swabs of horses with supposedly acute respiratory disease submitted to the GD laboratory during the SEIN reporting period described in this paper. EHV-1: equid alphaherpesvirus 1; EHV-4: equid alphaherpesvirus 4; EIV: equine influenza virus; *S. equi*: *Streptococcus equi* subsp. *equi*.

Year	EHV-1	EHV-4	EIV	*S. equi*
2019 ^1^	14/491 (2.9%)	19/334 (5.7%)	12/329 (3.6%)	84/538 (15.6%)
2020	23/813 (2.8%)	42/658 (6.4%)	23/632 (3.6%)	183/1081 (16.9%)
2021	40/3361 (12%)	55/1268 (4.3%)	13/1049 (1.2%)	289/1685 (17.2%)
2022	40/1012 (4.0%)	75/863 (8.7%)	14/826 (1.7%)	321/1546 (19.5%)
2023 ^2^	25/628 (4.0%)	33/307 (10.7%)	4/249 (1.6%)	62/438 (14.2%)
Total	142/6305 (2.3%)	224/3430 (6.5%)	66/3085 (2.1%)	939/5288 (17.8%)

^1^ June to December; ^2^ January to April.

**Table 3:** Reports for equid alphaherpesvirus 1 (EHV-1) according to disease syndrome. EHM: equine herpesvirus myeloencephalopathy.

Year	Respiratory/ Not Specified	Abortion/ Neonatal Mortality	EHM	Total
**2019 ^1^**	2	1	4	7
**2020**	4	9	3	16
**2021**	4	6	3	13
**2022**	2	4	3	9
**2023 ^2^**	1	2	0	3
**Total**	**13**	**22**	**13**	**48**

^1^ June to December; ^2^ January to April.

**Table 4:** Clinical signs in single respiratory equid alphaherpesvirus 1 (EHV-1), equid alphaherpesvirus 4 (EHV-4), equine influenza virus (EIV), or *Streptococcus equi* subsp. *equi* outbreaks reported within SEIN (June 2019–April 2023). For each clinical sign, different letters denote a significant difference (*p* < 0.05) between pathogens, as determined by Fisher’s exact test.

Clinical Signs	EHV-1 (*n* = 13)	EHV-4 (*n* = 50)	EIV (*n* = 19)	*S. equi* (*n* = 233)
Fever	100.0%	84.0%	78.9%	80.3%
Nasal discharge	69.2%	72.0%	68.4%	79.4%
Lymphadenopathy head and throat area	0.0% ^a^	18.0% ^b^	15.8% ^c^	39.9% ^a,b,c^
Coughing	23.1%	34.0% ^a^	52.6% ^b^	14.6% ^a,b^
Abscessation	0.0%	0.0% ^a^	0.0% ^b^	16.7% ^a,b^
Depression/lethargy	23.1% ^a^	8.0%	10.5%	3.9% ^a^
Stridor/dyspnea	0.0%	8.0%	0.0%	4.3%
Anorexia/weight loss	7.7%	8.0%	10.5%	3.9%
Limb edema	23.1% ^a,b^	2.0% ^a^	0.0%	0.9% ^b^
Neurological signs/ataxia	0.0%	2.0%	0.0%	0.9%
Other clinical signs	0.0%	6.0%	5.3%	1.3%

## Data Availability

No new data were created.

## Abbreviations

The following abbreviations are used in this manuscript:

EFPB	Equi Focus Point Belgium
EHM	Equine herpesvirus myeloencephalopathy
EHV-1	Equid alphaherpesvirus 1
EHV-2	Equid gammaherpesvirus 2
EHV-4	Equid alphaherpesvirus 1
EHV-5	Equid gammaherpesvirus 5
EIV	Equine influenza virus
ERAV	Equine rhinitis A virus
ERBV	Equine rhinitis B virus
KNMvD	Royal Veterinary Association of the Netherlands (*Koninklijke Nederlandse Maatschappij voor Diergeneeskunde*)
PCR	Polymerase chain reaction
RESPE	Réseau d’Epidémio-Surveillance en Pathologie Equine
*S. equi*	*Streptococcus equi* subsp. *equi*
*S. zooepidemicus*	*Streptococcus equi* subsp. *zooepidemicus*
SEIN	Surveillance of Equine Infectious diseases in the Netherlands
